# A Comprehensive Analysis Identified Hub Genes and Associated Drugs in Alzheimer's Disease

**DOI:** 10.1155/2021/8893553

**Published:** 2021-01-09

**Authors:** Qi Jing, Hui Zhang, Xiaoru Sun, Yaru Xu, Silu Cao, Yiling Fang, Xuan Zhao, Cheng Li

**Affiliations:** ^1^Department of Anesthesiology, Shanghai Tenth People's Hospital, Tongji University School of Medicine, Shanghai 200072, China; ^2^Department of Anesthesiology and Perioperative Medicine, Shanghai Fourth People's Hospital Affiliated to Tongji University School of Medicine, Shanghai 200434, China

## Abstract

Alzheimer's disease (AD) is the most common neurodegenerative disease among the elderly and has become a growing global health problem causing great concern. However, the pathogenesis of AD is unclear and no specific therapeutics are available to provide the sustained remission of the disease. In this study, we used comprehensive bioinformatics to determine 158 potential genes, whose expression levels changed between the entorhinal and temporal lobe cortex samples from cognitively normal individuals and patients with AD. Then, we clustered these genes in the protein-protein interaction analysis and identified six significant genes that had more biological functions. Besides, we conducted a drug-gene interaction analysis of module genes in the drug-gene interaction database and obtained 26 existing drugs that might be applied for the prevention and treatment of AD. In addition, a predictive model was built based on the selected genes using different machine learning algorithms to identify individuals with AD. These findings may provide new insights into AD therapy.

## 1. Introduction

Alzheimer's disease (AD) is the most common neurodegenerative disease among the elderly and has become a growing global health problem of great concern [1]. The most typical clinical manifestations of AD are progressive memory loss and cognitive function decline. Currently, there are approximately 47 million individuals who suffer from dementia across the globe, and the number is expected to increase to 100 million by 2050 [2]. AD is undoubtedly the most prevalent form of dementia. The significant development among societies, the increased rate of ageing, and the increased life expectancy of the population have contributed to the steady increase in the prevalence of AD.

At the early stage, the most characteristic symptoms of AD are mild memory loss and fatigue, anxiety, or negative emotions. Then, the memory impairment is aggravated and the logical thinking and comprehensive analysis abilities decrease. As the condition worsens, the cognitive impairment becomes more serious and widespread, making the person incapable of simple daily life tasks such as dressing and eating; at this time, the individual may be diagnosed with AD dementia. Later in the disease, patients suffer from impaired mobility, hallucinations, and seizures. The average duration from symptom onset to death is 8.5 years [3]. At present, the underlying mechanism of AD is unclear and may be associated with pathological processes such as the deposition of extracellular amyloid-*β* (A*β*) plaques and intracellular neurofibrillary tangles in the brain [4, 5]. AD pathology is confirmed in the entorhinal and temporal cortexes. A previous study identified that the expression of genes highly correlates with AD tau pathology and is most significantly increased in the entorhinal cortex, followed by the temporal cortex [6]; tau pathology usually begins in the medial temporal lobe (entorhinal cortex and hippocampus) in the allogeneic cortex. It is generally believed that the entorhinal cortex is the earliest brain structure with pathological changes in AD, while layer II of the entorhinal cortex is one of the most severely affected structures in AD [7, 8]. Furthermore, the brains of AD patients also show greater volume loss in the entorhinal cortex [9]. However, no specific therapeutics are currently available to provide the sustained remission of AD.

Traditionally, the glutamatergic system is considered the major factor affecting AD progression. All currently approved clinical drugs for AD are modulators, targeting cholinergic and glutamatergic systems, but they do not lead to the sustained remission of AD. Evidence suggests that modifying risk-added lifestyles and initiating drug and nondrug therapies in the early stage of the disease help maintain self-care ability and significantly reduce the burden of disease management. However, these changes do not alter the outcome of the disease [10]. Therefore, early AD identification and intervention are top priorities worldwide. As revealed in recent years, bioinformatics plays an important role in disease diagnosis and treatment [11].

**Figure 1:**
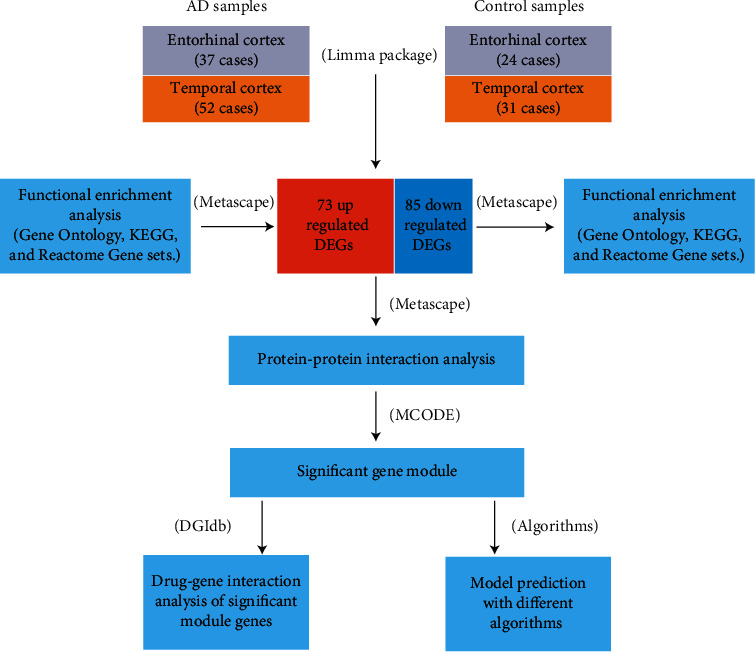
An overview workflow of this study.

In this study, we used comprehensive bioinformatics to determine the potential genes whose expression levels were different between the entorhinal and temporal lobe cortex samples from cognitively normal individuals and patients with AD. Then, we clustered these genes for the protein-protein interaction (PPI) analysis and identified significant genes that had more biological functions. Besides, we conducted the drug-gene interaction analysis of module genes using the drug-gene interaction database (DGIdb), which might contribute towards matching some existing drugs and subsequently finding alternatives for the prevention and treatment of AD. In addition, a predictive model was built based on the selected genes using different machine learning algorithms to identify individuals with AD. The workflow of the analysis is schematically shown in Figure 1.

## 2. Materials and Methods

### 2.1. Microarray Data Analysis

GSE118553 expression profiles and related clinical information data were retrieved and obtained from the NCBI-GEO website (https://www.ncbi.nlm.nih.gov/geo/) [6]. Entorhinal tissue samples (37 AD and 24 control samples) and temporal tissue samples (52 AD and 31 control samples) were included in the dataset. The corresponding GPL10558 platform annotation file included more than 31,000 annotated genes with more than 47,000 probes that were applied to convert the probes into target gene samples. If the target gene was annotated with two or more probes, the mean value was calculated. Among the targeted genes, the protein-coding genes were selected by referring to the human genome assembly *GRCh38*. Then, the Limma package [12] for the R environment was used to detect the differentially expressed genes (DEGs) between the AD and control samples of both entorhinal and temporal cortex tissues. DEGs were screened with the following cut-off criteria: [log_2_ fold change (FC)] > 0.5 and *P* value < 0.05. Overlapping DEGs between two brain regions were obtained using the Venn diagram packages [13, 14] in the R environment.

### 2.2. DEG Functional Enrichment Analysis

Gene enrichment analysis of DEGs was performed on the web-based portal Metascape (http://metascape.org/) [15], using the Gene Ontology biological process and the Kyoto Encyclopedia of Genes and Genomes (KEGG) pathway. The top ten enrichment terms were visualized using ggplot2 [16] package in R.

### 2.3. Protein-Protein Interaction Enrichment Analysis

For all the DEGs, PPI network analysis was conducted on Metascape using the following databases: BioGrid [17], InWeb_IM [17], and OmniPath [18]. In addition, if the network contained 3–500 proteins, the Molecular Complex Detection (MCODE) algorithm was applied to identify densely connected network components [18]. Pathway and process enrichment analyses were applied to each MCODE component independently, and the three best-scoring terms (based on the *P* value) were retained as the functional description of the corresponding components. Genes in each MCODE analysis were identified as potential target genes in AD prognosis and used for drug-gene interaction analysis and predictive model construction.

### 2.4. Drug-Gene Interaction Analysis

To explore the potential applications of the existing AD drugs, we designed an interactive model to identify interactions between genes and the existing drugs. Module genes were substituted into the drug-gene database (DGIdb: https://www.dgidb.org) [19] as potential targets to search for existing agonists or inhibitors. The FDA-approved drugs with antagonist or agonist functions were screened, and the interactions between the selected drugs and corresponding target genes were visualized in Cytoscape (version 3.7.1) [20].

### 2.5. Model Prediction

To explore whether MCODE genes have a function in the identification of AD samples, we built a prediction model using several machine learning algorithms depending on MCODE genes. Support Vector Machines (SVM) [21], Decision Tree [22], Random Forest [23], *K*-Nearest Neighbors (KNN) [24], and Naïve Bayes [25] were used. Considering the small sample size of this study, dividing the data into a training set, test set, and validation set was not appropriate. Therefore, to make the best use of the data, we applied a fivefold cross-validation method, which divided the data into five mutually exclusive subsets of similar size [26]. One of the subsets was selected as the test set and the other four subsets were used as the training set. Subsequently, five different results were obtained; finally, the average of the five test results was obtained. We used model evaluation indexes, such as accuracy, precision, recall, F1 score, and area under the curve (AUC) which were calculated as the evaluation matrices for the model. The model with the best performance was selected and deemed to have the ability to predict individuals with AD; if the performance of any two models was similar, the model with the larger AUC was considered as the best one. The AUC was used as a quantitative measure of the model quality, which was classified as poor (0.5–0.6), average (0.6–0.7), good (0.7–0.8), very good (0.8–0.9), and excellent (0.9–1). A better model was indicated with a higher AUC value, and a perfect model was indicated by an AUC value of 1 [1, 27]. Both model building and model performance assessment were performed using the Scikit-Learn library, which contains multiple machine learning algorithms in Python.

## 3. Results

### 3.1. DEG Identification

**Figure 2:**
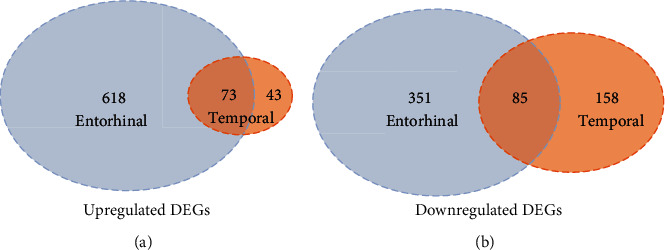
Overlapping DEGs across the entorhinal cortex and the temporal cortex. (a) 73 overlapping upregulated DEGs across the entorhinal cortex and the temporal cortex. (b) 85 overlapping downregulated DEGs across the entorhinal cortex and the temporal cortex.

The differential expression analysis showed 691 upregulated and 636 downregulated genes in the entorhinal cortex that were detected based on the following cut-off criteria: ∣log_2_ fold change (FC) | >0.5 and *P* value < 0.05, as well as 116 upregulated and 243 downregulated genes that were identified in the temporal lobe cortex. Among the DEGs, 158 overlapping DEGs present in both regions of the brain were obtained using the Venn diagram package, including 73 upregulated and 85 downregulated genes (Figure 2, Table 1).

### 3.2. Functional Enrichment Analysis of DEGs

**Figure 3:**
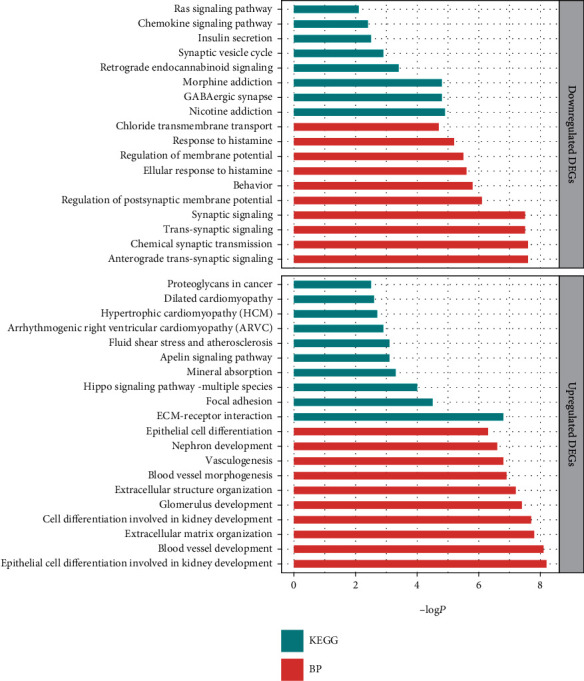
Top 10 BP and KEGG analysis terms of overlapping DEGs. BP: biological process; KEGG: Kyoto Encyclopedia of Genes and Genomes.

To outline GO and functional enrichments of overlapping DEGs, we applied Metascape and executed BP annotation and KEGG analysis of 73 overlapping and upregulated and 85 overlapping and downregulated DEGs, respectively. The top ten most significant results are shown in Figure 3, except for the downregulated DEGs only enriched in eight terms of the pathways. In the BP category, downregulated genes were mainly involved in anterograde transsynaptic signaling, chemical synaptic transmission, and transsynaptic signaling; upregulated genes were enriched in epithelial cell differentiation involved in kidney development, blood vessel development, and extracellular matrix organization. With regard to KEGG signaling pathway enrichment, downregulated genes were mainly related to nicotine addiction, GABAergic synapse, and morphine addiction; upregulated genes were significantly involved in ECM-receptor interaction, focal adhesion, and Hippo signaling pathways.

### 3.3. Protein-Protein Interaction Enrichment Analysis

**Figure 4:**
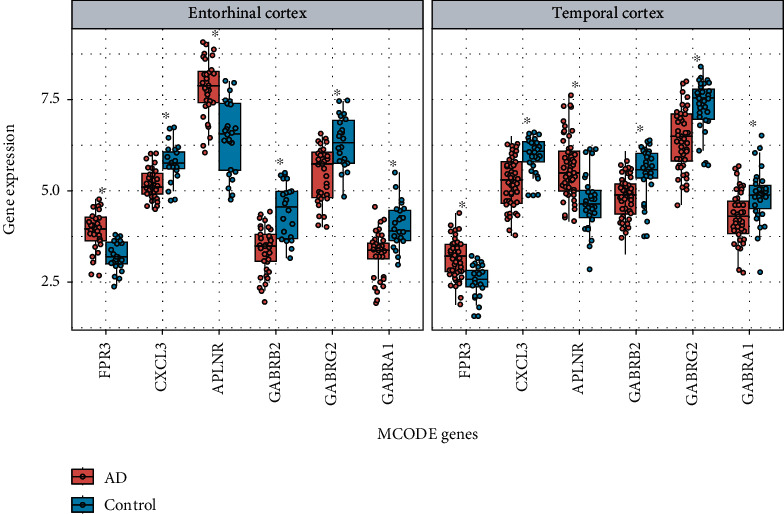
Expression of 6 MCODE genes in the entorhinal cortex and the temporal cortex, respectively (^∗^*P* < 0.05).

PPI analysis of DEGs was performed in Metascape [15], and two significant gene modules were selected using the MCODE application; each module consisted of three MCODE genes (Table 2). The genes in MCODE_1 were significantly enriched in peptide ligand-binding receptors, class A/1 (rhodopsin-like receptors), and G alpha (i) signaling event processes. The MCODE_2 genes were significantly enriched in the GABA-A receptor and cellular response to histamine processes. The expression of the six MCODE genes in the entorhinal and temporal cortexes is displayed in Figure 4; it contained two upregulated (*FPR3* and *APLNR*) and four downregulated genes (*CXCL3*, gamma-aminobutyric acid type A receptor subunit beta 2 (*GABRB2*), gamma-aminobutyric acid type A receptor subunit gamma 2 (*GABRG2*), and gamma-aminobutyric acid type A receptor subunit alpha 1 (*GABRA1*)).

### 3.4. Drug-Gene Interaction Analysis

**Figure 5:**
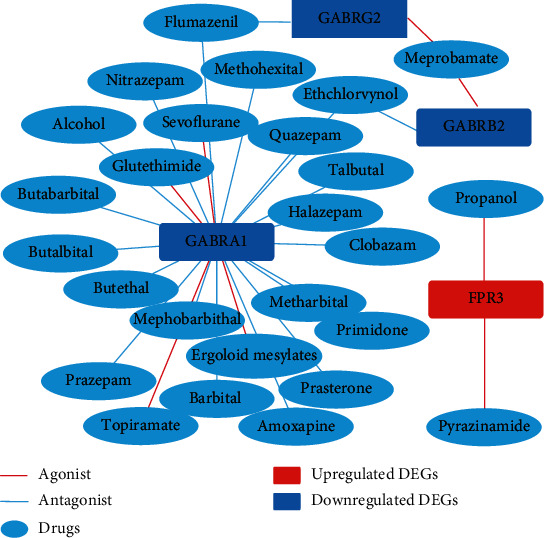
Drug-gene interaction.

The six MCODE genes clustered in the significant gene module were selected to perform drug-gene interaction analysis, which was aimed at looking for FDA-approved agonists and antagonists in the DGIdb database. We found that there were four target genes to 29 potential existing drugs. Moreover, 3 undefined drugs were removed, and 26 drugs that were agonists or antagonists were obtained, including two for formyl peptide receptor 3 (*FPR3*), 23 for *GABRA1*, two for *GABRB2*, and two for *GABRG2*. Psychiatric drugs with known indications accounted for the majority. We found that among the obtained drugs, ethchlorvynol and flumazenil act on *GABRB2* and *GABRG2*, respectively; both also act on *GABRA1*. Meprobamate acts on both *GABRB2* and *GABRG2* (Figure 5, Table 3).

### 3.5. Model Prediction

**Figure 6:**
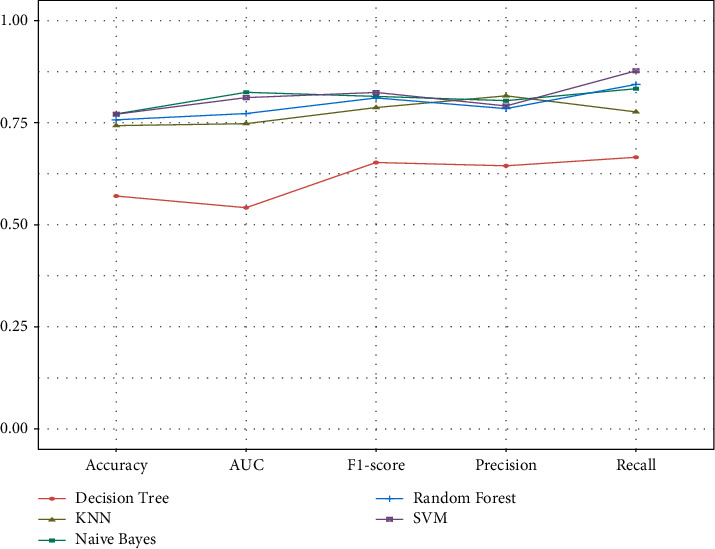
Prediction results based on different algorithms.

In total, 144 results for the gene expression in brain tissues were selected for the model construction using five algorithms and tested using fivefold cross-validation. The performance of the models is displayed in Table 4 and Figure 6. The fivefold cross-validation test showed that the SVM, Naïve Bayes, and Random Forest algorithms performed well. Then, we compared the uniformity of each algorithm's AUC in its category and chose the best performing model. The Naïve Bayes predictive model showed the highest AUC (82.45%) compared to the other two models (SVM: 81.15%, Random Forest: 77.25%), indicating that it had a good capability of predicting individuals with AD.

## 4. Discussion

AD is a common dementia with the highest fatality among the elderly, and the incidence of this disease shows a positive correlation trend with the patient's age. Age, gender, and genetics are unregulated factors that affect the occurrence of AD. Genetics plays an important role in the occurrence of AD. Presenilin 1, presenilin 2, and the amyloid precursor protein were identified to contribute or to be responsible for family AD [28]. Amyloid plaques, tau tangles, and neuron loss are characteristics of the AD brain [4], but the molecular changes underpinning these pathological features have not been fully elucidated. In recent years, transcriptomics has played an important role in revealing the pathogenesis of the disease and finding targeted drugs. Revealing the characterization of transcriptional alterations of the brain during disease development might offer some insights into the pathogenesis of AD. The purpose of this study was to discover potential mechanisms and hub genes in AD prognosis through the analysis of the transcriptional alteration in the entorhinal and temporal cortexes between AD and normal samples using bioinformatics methods.

In this study, according to the gene-drug interaction analysis, we found 26 potential drugs for AD treatment, which target four genes (*FPR3*, *GABRB2*, *GABRG2*, and *GABRA1*). FPR3, also known as FPRL2, is a member of the FPR family localized within the cytoplasm [29]. Human FPRs belong to the G protein-coupled chemoattractant receptors, which are expressed in blood innate immune cells, including neutrophils, monocytes, and natural killer (NK) cells, playing an important role in infection and inflammation. Interestingly, it has been reported that another subtype of the FPR family, FPRL1, can be specifically activated by *Αβ*_42_, suggesting that FPRL1 may be involved in the pathological process of neurodegenerative diseases such as AD [30]. *GABRB2* encodes the *β*2 subunit of the gamma-aminobutyric acid type A (GABA-A) receptor, which regulates the intracellular Ca^2+^ concentration and plays an important role in the nervous system [31]. Some researchers believe that GABRB2 is related to schizophrenia [32, 33], but this view is widely controversial. *GABRG2* encodes the GABA-A receptor subunit *γ*2. Mutation of this gene contributes to the pathogenesis of both febrile seizures and childhood absence epilepsy [34]. *GABRA1* encodes the GABA-A receptor subunit *α*1. It is confirmed that *GABRA1* mutation predisposes humans towards a common idiopathic generalized epilepsy syndrome [35].

GABA is the main inhibitory neurotransmitter in the mammalian brain, while the GABA-A receptor is the multi-subunit chloride channel that mediates the fastest inhibitory synaptic transmission in the central nervous system. The genes selected through our research are closely related to the GABA-A receptor. *β*2, *γ*2, and *α*1 subunits, which are encoded by *GABAB2*, *GABAG2*, and *GABRA1*, respectively, are the most abundant receptor forms (*α*1*β*2*γ*2) in the brain. Patients with AD exhibit nonamnestic manifestations, such as depression, anxiety, and sleep disorders, which may be attributed to GABAergic dysfunction [36]. The balance of excitatory and inhibitory signaling governs the function of the nervous system. The destruction of GABAergic neurons and GABA receptors disrupts the excitatory/inhibitory (E/I) balance, which is a crucial mechanism involved in epilepsy and seizures. The seizure rate of patients with AD significantly increases compared with that of normal people [37], and the stability of the neural network in the AD brain is decreased, suggesting that the E/I imbalance is strongly related to the pathogenesis of AD. Researchers have studied the pathogenic factors *Aβ*, *BACAE1*, and *APOEε4* [38] and hyperactive glial cells [39] and concluded that all result in GABAergic dysfunction and E/I imbalance in AD mouse models [40]. More importantly, correcting the E/I imbalance improves the cognitive dysfunction in mice with AD. Besides, ageing is the strongest risk factor for AD and is related to GABAergic damage, which may lead to cognitive decline in rodents and primates. The prevalence of AD in females is higher than that in males; further, there are obvious sex-based differences in GABAergic signaling and progression of AD. These findings indicate that GABAergic dysfunction may be involved in AD pathogenesis and our work supports this view.

Traditionally, the GABAergic system is believed to be relatively conserved throughout AD progression, while the dysfunction of the glutamatergic system is considered as the major factor responsible for AD. Currently approved clinical drugs for AD are modulators of the cholinergic and glutamatergic targets, but their effects are limited, suggesting that other drugs are needed to restore the E/I imbalance. Presently, the GABAergic dysfunction is thought to be a significant cause of E/I imbalance and pathogenesis in the AD brain, making it a potential therapeutic target. The GABA-A receptor has already been identified as a prolific target for some therapeutic drugs, including benzodiazepines, barbiturates, anesthetics, and ethanol [41]. It has been found that a low dose of benzodiazepine clonazepam (0.05 mg/kg) is beneficial to AD [42], and a daily peritoneal injection of the GABA-A receptor potentiator pentobarbital sodium rescues the learning and memory impairment in ApoE4-Ki mice, while the GABA-A receptor antagonist reverses this rescue [30]. To restore the E/I balance, five agonists (meprobamate, topiramate, glutethimide, sevoflurane, and ergoloid mesylates) targeting the GABA-A receptor are useful in AD, while the rest of the antagonists may be related to the aggravation of the cognitive impairment. Therefore, our work may have important indications in the use of these drugs from a new perspective.

To detect the predictive function in identified AD samples based on the selected MCODE genes and build the predictive model, five different algorithms usually used in machine learning to solve supervised binary classification problems were applied. In total, results from the expression of 144 genes in brain tissues were used for model establishment. According to the 5-fold cross-validation method results, the SVM, Naïve Bayes, and Random Forest algorithms performed well. Among the models, the AUC of the Naïve Bayes algorithm in AD classification was superior to that of the other methods, indicating that this model may be applied in AD diagnosis. It also implied that the MCODE genes might play a critical role in AD prognosis.

## 5. Conclusions

In conclusion, we obtained six hub genes (*FPR3*, *CXCL3*, *APLNR*, *GABRB2*, *GABRG2*, and *GABRA1*) and 26 FDA-approved existing drugs through the application of an integrated bioinformatics approach. These findings may provide new insights into AD therapy. The risk prediction model we have established can be applied to the early screening of high-risk populations and provide disease management and drug intervention in the early stage. This strategy may significantly delay the development of AD, improve the quality of life of AD patients, and reduce the social burden associated with such conditions. We expect to conduct molecular experiments and clinical trials to confirm the results of this research.

## Figures and Tables

**Table 1:** 158 DEGs both in the entorhinal cortex and the temporal cortex.

DEGs	Gene name
Upregulated DEGs (73)	C1QTNF5, ITGB4, SYTL4, LAMB2, ANTXR2, COLEC12, TNS3, PPIC, C4B, INS-IGF2, ABCC4, SLC15A3, YAP1, DUSP1, FAM89A, ITPKB, NWD1, ASCL1, NOTCH2, AEBP1, CHST6, QPRT, DDIT4L, AKR1C3, IPO8, EPS8, WWTR1, PCDH18, GAS1, DSE, DCN, DST, EZR, SCARA3, TEAD2, BACE2, APLNR, COL6A3, LTBP1, IL13RA1, ITGB8, NOTCH3, FPR3, CDC14A, PLXNB1, RASL12, EMP3, ACTA2, PGR, CD44, PIPOX, HEPH, SLC13A4, BDH2, GEM, MT1F, HHAT, ERMAP, ITGA10, CCL2, RIN2, SERPINA3, GPR4, GFAP, EDNRA, KLF2, CAV1, TTR, SRGN, MYH11, SALL3, FOXQ1, MT1H

Downregulated DEGs (85)	GAS7, TAGLN3, RPH3A, SYNGR1, PRKCZ, RASGRF1, KLC1, MICAL2, NEFM, SEZ6L2, VSNL1, COQ4, CRYM, NEFL, GABRB2, SVOP, RPRML, PJA1, SULT4A1, BBS9, DOT1L, ABCC8, TSPY3, ITIH3, TNKS2, PIGV, ATP6V1G2, TMEM174, HPRT1, CXCL3, CORO6, CLMN, RPL36A, PDIA2, DYRK2, GRK4, FGF12, GABRA1, CYP4X1, SCN2A, CDKN2C, UBE2J2, MDK, SNAP25, CKMT1B, GAK, GABRG2, ARRDC2, SCN2B, R3HDM1, CAMTA1, PCDH10, TUBB3, FBXO2, FOXE3, C10orf82, USP20, KRT32, CLEC2L, SH3GL2, CYP1A1, PAK1, KLK11, NRXN3, FGF9, PCSK1, PNMA3, VIP, RGS7, OPCML, INA, ADCYAP1, RGS4, NUDT10, SPHKAP, ST8SIA3, MYT1L, GAD1, ANO3, GPR26, GABRD, NCALD, EIF1AY, CHGB, SYT1

**Table 2:** Three best-scoring pathway and process enrichment analysis terms of each MCODE component.

MCODE	Genes	GO	Description	log_10_(*P*)
MCODE_1	FPR3 CXCL3 APLNR	R-HSA-375276	Peptide ligand-binding receptors	-6.3
		R-HSA-373076	Class A/1 (rhodopsin-like receptors)	-5.6
		R-HSA-418594	G alpha (i) signaling events	-5.3

MCODE_2	GABRB2 GABRG2 GABRA1	CORUM:7461	GABA-A receptor (GABRA1, GABRB2, and GABRG2)	-12.4
		CORUM:5809	GABA-A receptor (GABRA1, GABRB2, and GABRG2)	-12.4
		GO:0071420	Cellular response to histamine	-10.6

**Table 3:** Gene-drug interaction information by mapping in the DGIdb database.

Gene	Drugs	Interaction	Gene	Drugs	Interaction
FPR3	Propanol	Agonist	GABRA1	Prasterone	Antagonist
FPR3	Pyrazinamide	Agonist	GABRA1	Butethal	Antagonist
GABRB2	Ethchlorvynol	Antagonist	GABRA1	Mephobarbital	Antagonist
GABRB2	Meprobamate	Agonist	GABRA1	Clobazam	Antagonist
GABRG2	Meprobamate	Agonist	GABRA1	Sevoflurane	Agonist
GABRG2	Flumazenil	Antagonist	GABRA1	Methohexital	Antagonist
GABRA1	Topiramate	Agonist	GABRA1	Metharbital	Antagonist
GABRA1	Halazepam	Antagonist	GABRA1	Alcohol	Antagonist
GABRA1	Amoxapine	Antagonist	GABRA1	Primidone	Antagonist
GABRA1	Butalbital	Antagonist	GABRA1	Ergoloid mesylates	Agonist
GABRA1	Talbutal	Antagonist	GABRA1	Nitrazepam	Antagonist
GABRA1	Quazepam	Antagonist	GABRA1	Butabarbital	Antagonist
GABRA1	Glutethimide	Agonist	GABRA1	Flumazenil	Antagonist
GABRA1	Prazepam	Antagonist	GABRA1	Barbital	Antagonist
GABRA1	Ethchlorvynol	Antagonist			

**Table 4:** Prediction results based on different algorithms.

Algorithms	Accuracy (%)	Precision (%)	Recall (%)	F1 score (%)	AUC (%)
Naive Bayes	77.14	80.41	83.33	81.45	82.45
SVM	77.07	78.11	87.71	82.42	81.15
Random Forest	75.71	78.46	84.38	81.07	77.25
KNN	74.29	81.59	77.65	78.72	74.77
Decision Tree	57.04	64.45	66.54	65.26	54.18

## Data Availability

The data used to support the findings of this study are from previously reported studies and datasets, which have been cited.
